# Supplementary material for: Cabazitaxel-Loaded Nanocarriers for Cancer Therapy with Reduced Side Effects
Source: Pharmaceutics. 2019 Mar 25;11(3):141. doi: 10.3390/pharmaceutics11030141 (PMC6470818; doi:10.3390/pharmaceutics11030141)
Supplement: Supplementary file 1 [file pharmaceutics-11-00141-s001.pdf]

pharmaceutics

MDPI

Supplementary Materials: Cabazitaxel-Loaded Nanocarriers for Cancer Therapy with Reduced Side Effects

Nagavendra Kommineni, Shaheen Mahira, Abraham J. Domb and Wahid Khan

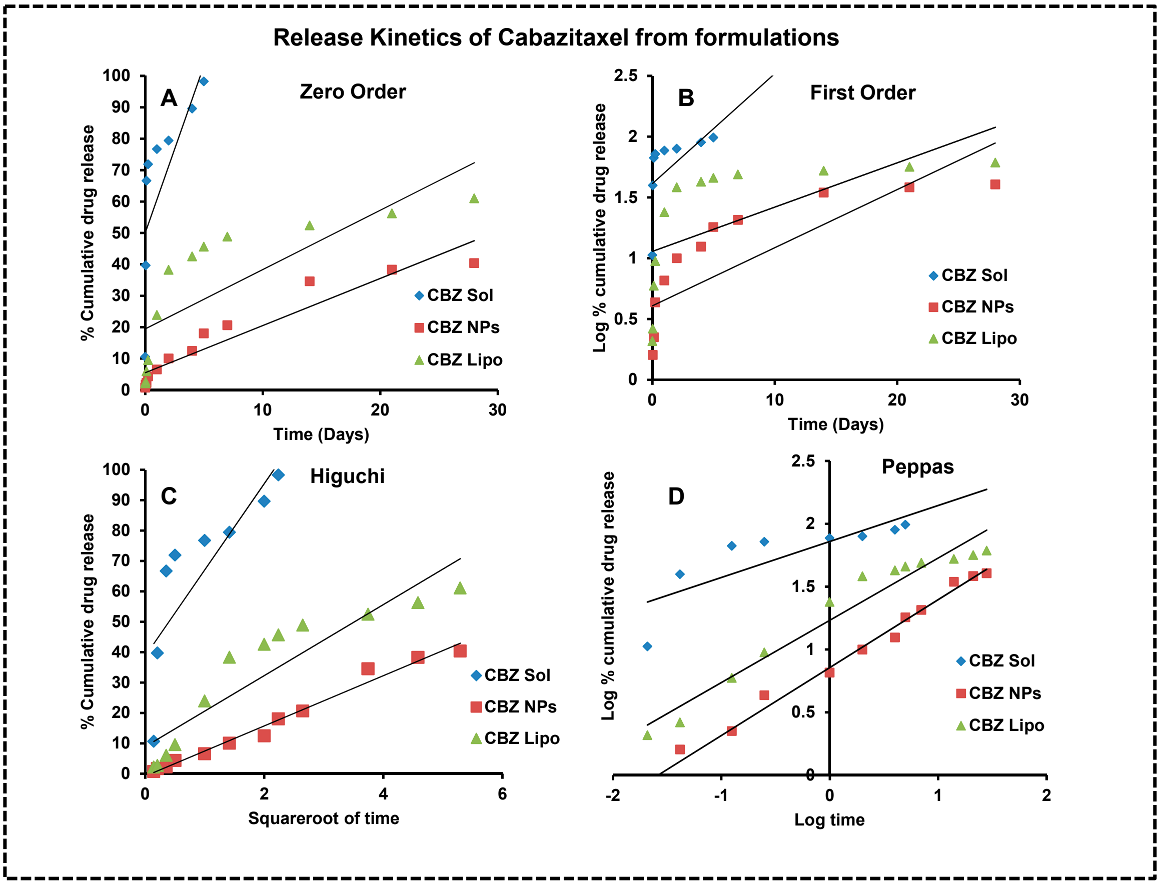

Figure S1. Cabazitaxel release kinetics from different formulations of CBZ Sol (cabazitaxel micellar solution), CBZ NPs (cabazitaxel nanoparticles), and CBZ Lipo (cabazitaxel liposomes): (**A**) zero-order model, (**B**) first-order model, (**C**) Higuchi model, and (**D**) Peppas model.
